# Analysis of the efficacy and adverse effects of nusinersen in the treatment of children with spinal muscular atrophy in China

**DOI:** 10.1002/brb3.3630

**Published:** 2024-07-21

**Authors:** Dan Li, Jie Yang, Xueying Wang, Lin Yang, Rong Luo, Shaoping Huang

**Affiliations:** ^1^ Department of Pediatrics The Second Affiliated Hospital of Xi'an Jiaotong University Xi'an Shaanxi China; ^2^ Department of Pediatrics West China Second University Hospital Sichuan University Chengdu Sichuan China

**Keywords:** clinical effect, motor function, nusinersen, SMA

## Abstract

**Objective:**

This study was based on a retrospective clinical observational cohort study of a two‐center application of nusinersen in China to evaluate the clinical efficacy and adverse effects of nusinersen in the treatment of SMA (spinal muscular atrophy) Types 1–3.

**Methods:**

Clinical data from children with clinically and genetically confirmed 5qSMA from a double center in western China (the Second Affiliated Hospital of Xi'an Jiaotong University and the Second Hospital of West China of Sichuan University). All children were younger than 18 years of age. Patients were assessed for motor function and underwent blood and fluid tests before each nusinersen injection.

**Results:**

At 14‐month follow‐up, 100% of children had improved their HFMSE (Hammersmith Functional Motor Scale Expanded) score, 83.6% had improved their CHOP INTEND (Children's Hospital of Philadelphia Infant Test of Neuromuscular Disorders) score, and 66.6% had improved their RULM (Revised Upper Limb Module) score by ≥3 points from baseline, and their 6MWT (6‐min walk test) was 216.00 ± 52.08 m longer than at baseline. The age of the child at the start of treatment was negatively correlated with the clinical efficacy of nusinersen; the younger the child, the better the response to treatment. No significant adverse effects affecting the treatment and quality of life of the child were observed during the treatment of SMA with nusinersen.

**Conclusion:**

This study concluded that nusinersen is clinically beneficial for children with SMA in western China, with mild adverse effects.

## Graphical abstract

Nusinersen is clinically beneficial for children with SMA types 1,2 and 3 in western China, with mild adverse effects.

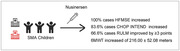

## 1 INTRODUCTION

Spinal muscular atrophy (SMA) is an autosomal recessive disorder caused by low levels of the survival of motor neuron (SMN) protein (Lefebvre et al., 1995; Wirth et al., 2020). It is characterized by progressive muscle atrophy and weakness (Prior et al., 2021) and has a prevalence of 1 in 11,000 (Sugarman et al., 2012; Wirth, 2021). SMA used to be the main monogenic disease causing infant mortality. SMA is clinically classified into four types based on the patient's age of onset and the best motor milestone event ever reached (Cho & Dreyfuss, 2010; Zhao et al., 2022). SMA Type 1 refers to children who present with symptoms in the first 6 months of infancy and never achieve the ability to sit unaided without support, once known as Werdnig–Hoffmann disease, which accounts for approximately 60% of all SMA patients (El‐Matary et al., 2004; Lowes et al., 2019). Children with SMA Type 2, who present with weakness in late infancy and can achieve independent sitting but never walk independently, account for approximately 30% of SMA patients. Children with SMA Type 3 (Kugelberg–Welander disease) have the best motor functional status of being able to walk independently and account for approximately 10% of patients (Salort‐Campana & Quijano‐Roy, 2020). SMA Type 4 is a small group that does not show muscle weakness until adulthood (Sarv et al., 2021; Singh et al., 2021).

Despite a growing number of clinical trials over the past few years, there is currently no available cure for SMA.

Nusinersen, an intrathecal injection of antisense oligonucleotides, is the first effective gene‐modifying treatment for SMA, which is an important advance in disease treatment (Chiriboga, 2017). Nusinersen has now been approved by the FDA (Pechmann & Kirschner, 2017) and the EMA (European Medicines Agency, www.ema.europa.eu/) for the treatment of 5qSMA, and the situation of SMA treatment is improving (Finkel et al., 2017, 2021). Nusinersen, a 2′‐*O*‐methoxyethyl phosphate antisense oligonucleotide, which was used in both Phase II and Phase III clinical trials to treat children with SMA Type 1, has been demonstrated to improve clinical symptoms and life expectancy but was not effective in all children (Finkel et al., 2021). Darras et al. (2019) demonstrated a favorable safety profile of nusinersen in children with symptomatic infantile‐ and later‐onset SMA. The real‐world study of nusinersen in Croatian SMA patients found that it is an effective and safe treatment for pediatric patients with all types of SMA. However, SMA Type 3 patients who started nusinersen treatment in adulthood did not show significant benefits (Belančić et al., 2023). With limited data, nusinersen has been rapidly licensed for clinical use, and nusinersen entered the Chinese market in October 2019. However, there are no reliable data on the efficacy and safety of nusinersen sodium in the treatment of SMA in the western Chinese population.

This study is based on a two‐center retrospective observational clinical cohort study of the application of nusinersen sodium in western China, with the aim of evaluating the clinical efficacy and adverse effects of nusinersen in the treatment of SMA Types 1–3 in children in western China.

## 2 MATERIALS AND METHODS

Clinical data were retrospectively collected from October 2019 to February 2022 from patients with 5qSMA treated with nusinersen at a double center in western China, the Second Affiliated Hospital of Xi'an Jiaotong University and the Second West China Hospital of Sichuan University. The study was approved by the Ethics Committee of the Second Affiliated Hospital of Xi'an Jiaotong University (No. 2022244) and the Second West Hospital of Sichuan University (No. 2022‐199). Each patient signed an informed consent form for lumbar puncture and nusinersen sheath injection. For children with severe scoliosis, children with a Cobb's angle greater than 50°, and children who had undergone spinal fusion surgery, we performed ultrasound‐guided lumbar puncture intrathecal administration. The rest of the children were administered by sheath injection using the traditional lumbar puncture method.

Inclusion criteria were patients with clinical signs of muscle weakness and myasthenia and a genetically confirmed diagnosis of 5qSMA, patients with a genetic test showing homozygous deletion or a compound heterozygous mutation in exon 7 of SMN, and patients with a known SMN2 copy number.

Exclusion criteria are patients who have stopped treatment for various reasons, such as severe infection, financial hardship, and death; patients over 18 years of age; patients who have failed nusinersen treatment; and patients with incomplete clinical information. The criteria for failed nusinersen treatment are two or more consecutive decreases in Children's Hospital of Philadelphia Infant Test of Neuromuscular Disorders (CHOP INTEND) or Hammersmith Functional Motor Scale Expanded (HFMSE) scores compared to baseline (Carrera‐García et al., 2021; Gavriilaki et al., 2022).

Patients are classified into SMA types based on when the clinical symptoms appeared and the best motor function the child ever achieved: Type 1 (never could sit), Type 2 (can now or ever sit unsupported), and Type 3 (can now or ever walk unaided). To assess motor function, CHOP INTEND was used for all patients younger than 2 years of age and for all patients who could not sit independently, with a total score of 0–64 (Finkel et al., 2017). Patients who could sit unaided were assessed using the HFMSE with a total score of 0–66, and those who could sit at a table were assessed with the Revised Upper Limb Module (RULM) (Coratti et al., 2021) for upper limb function with a total score of 0–37. For walkers, a 6‐min walk test (6MWT) and HFMSE assessment were performed (Gavriilaki et al., 2022).

### 2.1 Dosing method

**FIGURE 1:**
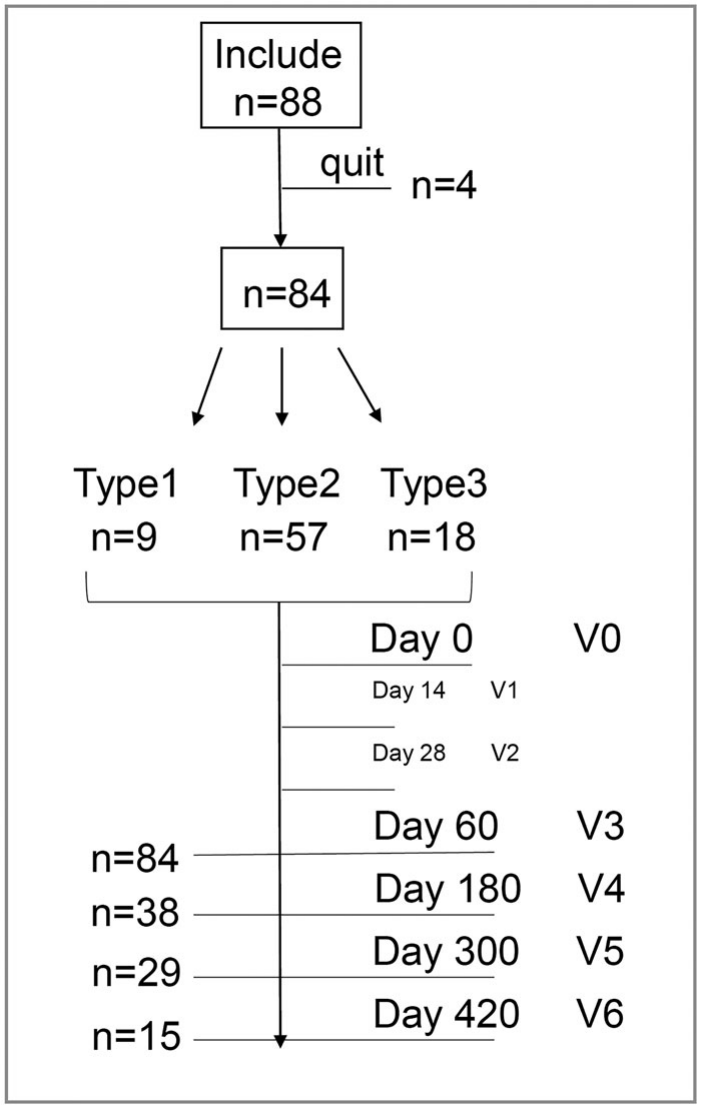
Workflow of the retrospective study. V, visit period.

Nusinersen is delivered intrathecally via the L3–4 or L2–3 intervertebral space at a consistent dose of 12 mg per patient, with the loading dose completed in the first 2 months in four sessions, on Days 1, 14, 28, and 63. Thereafter, the maintenance phase was entered, with sheath injections every 4 months. The first pre‐sheathing visit is considered V0, the baseline period, Day 14 visit is considered V1, Day 28 visit is considered V2, Month 2 visit is considered V3, Month 6 visit is considered V4, Month 10 visit is considered V5, Month 14 visit is considered V6, and so on, as shown in Figure 1.

### 2.2 Statistical analysis

The data were collated using EXCEL software and statistically analyzed using SPSS 19.0 software. The mean plus standard deviation was used for measurement data, and the number of cases and percentages were used for counting data. Comparisons between groups of measurement data were made using ANOVA, and correlations were analyzed using Spearman's test. Graphs were produced using GraphPad Prism 8 software. Statistical significance was considered at *p* < .05.

## 3 RESULTS

### 3.1 Demographic characteristics

A total of 88 SMA patients were treated with nusinersen sodium at a two‐center site in western China during the study period. Four people were excluded from the study: Two children with SMA Type 2 were sheathed twice and transferred to another treatment center for further treatment, and data were lost; one child with SMA Type 1 died after three treatments due to severe pulmonary infection, and one child with SMA Type 3 was treated four times and felt that the treatment was not effective and was transferred to another genetically modified treatment. Finally, 84 children were enrolled in the study. Fifteen (17.9%) completed 7 sessions, 29 (34.5%) completed the first 6 sessions, and 38 (45.2%) completed the first 5 sessions. A total of 84 (100%) children completed the first 4 sessions (Figure 1). A total of 46 children had varying degrees of scoliosis, and 1 had a spinal fusion prior to sheathing. Six of these patients, with severe scoliosis, Cobb's angle greater than 50°, and postoperative spinal fusion, were treated with ultrasound or CT‐guided sheathing of nusinersen sodium (Carrera‐García et al., 2021), all of which were successful. One patient had invasive ventilator‐assisted ventilation, seven had non‐invasive ventilator‐assisted ventilation, and one underwent gastrostomy at the end of loading volume therapy. The remaining patients did not require nasal feeding or assisted ventilation. Table 1 shows the clinical characteristics of all patients at baseline.

**TABLE 1:** Clinical characteristics of children with spinal muscular atrophy (SMA) Types 1–3.

	SMA1	SMA2	SMA3	Total
Number of patients, *n* (%)	9 (10.7)	57 (67.9)	18 (21.4)	84 (100)
Male/Female, *n*	3/6	34/23	6/12	43/41
Age at baseline (year, min–max)	4.64 (0.33–12)	6.52 (0.17,16.83)	7.10 (2,17)	6.47 (0.17,17)
Age of onset (year, min–max)	0.57 (0.02–1.08)	0.90 (0.17,2.00)	3.36 (0.75,15.00)	1.39 (0.02,15.00)
Age of genetic diagnosis (year, min–max)	1.07 (0.42–2.0)	2.53 (0.83,13.92)	6.03 (1.67,16)	3.14 (0.42,16)
Gene diagnosis to treatment delay time (year, min–max)	0.5 (0.00,1.3)	1.63 (0.00,13.52)	2.67 (0.00,14.5)	1.77 (0.00,13.52)
SMN2 copy number, *n* (%)				
2	3 (3.6)	8 (9.5)	0 (0.0)	11 (13.1)
3	5 (6.0)	50 (59.5)	17 (20.2)	72 (85.7)
4	0 (0.0)	0 (0.0)	1 (1.2)	1 (1.2)
Mutation type, *n* (%)				
Homozygous deletion	9 (10.7)	53 (63.1)	17 (20.2)	79 (94.0)
Compound heterozygous mutations	0 (0)	4 (4.8)	1 (1.2)	5 (6.0)
Baseline motor function score (min–max)				
CHOP INTEND	13.89 (4–34)	41.31 (8–60)	–	35.18 (4–60)
HFMSE score	–	13.71 (0–47)	42.28 (5–64)	20.99 (0–64)
RULM	–	20.10 (1–30)	29.53 (22–36)	22.72 (1–36)
6MWT (m)	–	–	171.38 (35–528)	171.38 (35–528)
Scoliosis, *n*	5	35	6	46
Assist ventilation, *n*	5	3	0	8
Gastrostomy, *n*	1	0	0	1
Ultrasound or CT‐guided sheath injection therapy, *n*	0	6	0	6

Abbreviations: 6MWT, 6‐min walk test; CHOP INTEND, Children's Hospital of Philadelphia Infant Test of Neuromuscular Disorders; HFMSE, Hammersmith Functional Motor Scales Expanded; RULM, Revised Upper Limb Module; SD, standard deviation; SMN, survival of motor neuron.

The mean age at onset of disease was 1.39 years in 84 patients, the mean age at initiation of treatment was 6.47 years, the mean age at genetic diagnosis was 3.14 years in all patients, and the delay between genetic diagnosis and initiation of treatment was 1.77 years. Nine patients (10.7%) had Type 1, 57 patients (67.9%) had Type 2, and 18 patients (21.4%) had Type 3. A total of 79 patients (94.0%) had homozygous deletions, and 5 patients (6.0%) had compound heterozygous mutations. The number of SMN2 copies ranged from 2 to 4, with 72 (85.7%) children having 3 copies and 11 (13.1%) having 2 copies. The CHOP INTEND score was performed in 35 (41.7%) children with scores ranging from 4 to 60, the HFMSE score was performed in 68 (86.1%) children with scores ranging from 0 to 64, the RULM score was performed in 53 (64.3%) children with scores ranging from 1 to 36, and the 6MWT test was performed in 16 (19.0%) children with scores of 35–528 m (Table 1).

### 3.2 HFMSE score

**FIGURE 2:**
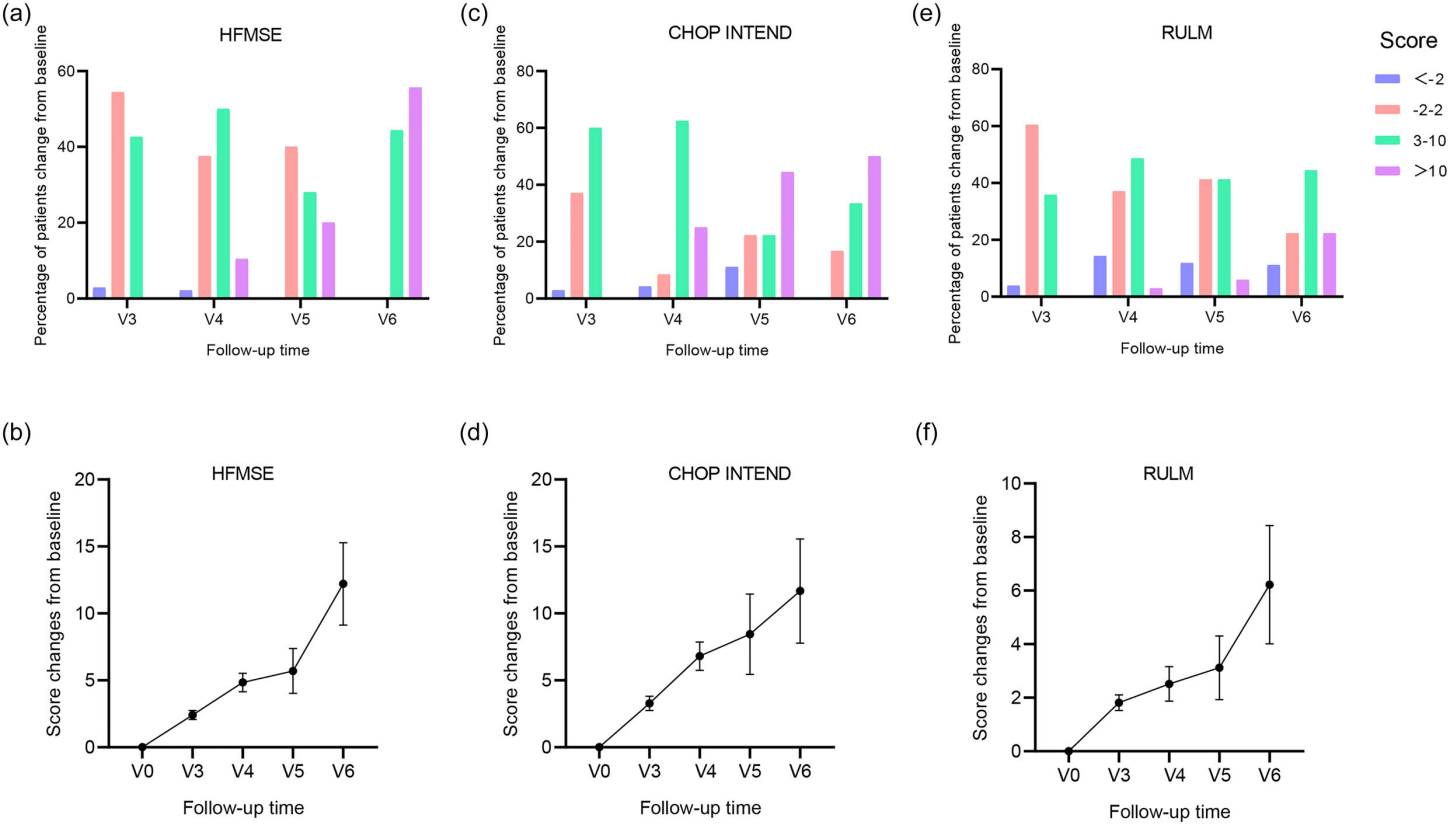
Changes in baseline and V3–V6 Hammersmith Functional Motor Scale Expanded (HFMSE) scores, Children's Hospital of Philadelphia Infant Test of Neuromuscular Disorders (CHOP INTEND) scores, and Revised Upper Limb Module (RULM) scores: (a) percentage of patients with different scores of changes from baseline in HFMSE scores during V3–V6; (b) change curve of HFMSE scores for baseline and V3–V6 period; (c) percentage of patients with different scores of changes from baseline in CHOP INTEND scores during V3–V6; (d) change curve of CHOP INTEND scores for baseline and V3–V6 period; (e) percentage of patients with different scores of changes from baseline in RULM scores during V3–V6; (f) change curve of RULM scores for baseline and V3–V6 period. V, visit period.

In this study, 68 children were scored on the HFMSE, with a mean score of 20.99 ± 16.95 at baseline and a V4 score of 22.86 ± 16.29 after treatment. One child (2.9%) had a V4 score below 2 from baseline, 18 children (37.5%) had a score between −2 and 2 from baseline, 24 children (50%) had a score above 2–10 from baseline, and 5 children (10.4%) had a score above 10 or more from baseline. HFMSE scores in V4–V6 visit periods change from baseline compared to V3 were significantly different; the proportion of children with scores higher than 2 increased gradually with the duration of treatment, and by V6, nine (100%) children had HFMSE scores ≥3 or more from baseline, with 55.6% of children with scores higher than 10 (Figure 2, Table 2).

**TABLE 2:** Changes in Hammersmith Functional Motor Scales Expanded (HFMSE) scores during different visit periods.

Follow‐up time	*N*	HFMSE mean ± SD	Change from baseline	Change from baseline (*n*, %)				*F* value	*p* value
				<−2	−2 to 2	3–10	>10		
V0	68	20.99 ± 16.95	–	–	–	–	–	–	–
V3	68	23.37 ± 17.11	2.40 ± 0.33	2 (2.9)	37 (54.4)	29 (42.6)	0 (0.0)	11.899	.000^a^
V4	48	22.86 ± 16.29	4.83 ± 0.69	1 (2.1)	18 (37.5)	24 (50)	5 (10.4)		
V5	25	23.68 ± 13.25	5.95 ± 1.73	0 (0.0)	10 (40)	7 (28)	5 (20)		
V6	9	33.90 ± 26.71	12.20 ± 3.07	0 (0.0)	0 (0.0)	4 (44.4)	5 (55.6)		

*Note*: V, visit.

^a^ Comparison change from baseline in V4–V6 with V3 period.

### 3.3 CHOP INTEND score

In this study, 35 children were measured with a CHOP INTEND score of 35.18 ± 18.34 at baseline. After nusinersen treatment, nine children had a CHOP INTEND score at V5, one child had a score of 2 below baseline, two children had a score between −2 and 2 at baseline, two children had a score of 3–10 above baseline, and four children had a score of 10 or more above baseline. There was a statistically significant difference among V4–V6 visits. The change from baseline to V3 was statistically significant. The change from baseline was greater as the duration of treatment increased, with CHOP INTEND improving by 11.66 ± 3.89 points from baseline in V6, and 83.6% of children had an improvement of ≥3 points in their assessment score from baseline (Figure 2, Table 3).

**TABLE 3:** Changes in Children's Hospital of Philadelphia Infant Test of Neuromuscular Disorders (CHOP INTEND) scores during different visit periods.

Follow‐up time	*N*	CHOP INTEND mean ± SD	Change from baseline	Change from baseline (*n*, %)				*F* value	*p* value
				<−2	−2 to 2	3–10	>10		
V0	35	35.18 ± 18.34	–					–	–
V3	35	38.06 ± 18.92	3.37 ± 0.54	1 (2.9)	13 (37.1)	21 (60)	0 (0.0)	5.466	.002^a^
V4	24	41.08 ± 17.55	7.08 ± 1.07	1 (4.2)	2 (8.4)	15 (62.5)	6 (25.0)		
V5	9	42.44 ± 19.98	8.44 ± 3.00	1 (11.1)	2 (22.2)	2 (22.2)	4 (44.4)		
V6	6	47.17 ± 19.88	11.66 ± 3.89	0 (0.0)	1 (16.7)	2 (33.4)	3 (50)		

*Note*: V, visit.

^a^ Comparison change from baseline in V4–V6 with V3 period.

### 3.4 RULM score

In this study, 53 children performed RULM score, with a mean score of 22.72 ± 8.57 at baseline. After nusinersen treatment, the RULM score improved by 3.12 ± 1.18 points from baseline by V5 stage. Seventeen children had a RULM score at the V5 visit, two children had a score of 2 below baseline, seven children had a score between −2 and 2 at baseline, seven children had a score of 3–10 above baseline, and one child had a score of 10 or more above baseline. There was an improvement of 6.22 ± 2.21 points at V6 compared to baseline, with 66.6% of children scoring ≥3 points better than baseline (Figure 2, Table 4).

**TABLE 4:** Changes in Revised Upper Limb Module (RULM) scores during different visit periods.

Follow‐up time	*N*	RULM mean ± SD	Change from baseline	Change from baseline (*n*, %)				*F* value	*p* value
				<−2	−2 to 2	3–10	>10		
V0									–
V3	53	22.72 ± 9.57	1.81 ± 0.29	2 (3.8)	32 (60.4	19 (35.8)	0 (0.0)	3.906	.011^a^
V4	35	23.26 ± 9.62	2.51 ± 0.64	5 (14.3)	13 (37.1)	17 (48.6)	1 (2.9)		
V5	17	20.71 ± 11.07	3.12 ± 1.18	2 (11.8)	7 (41.2)	7 (41.2)	1 (5.9)		
V6	9	23.56 ± 14.34	6.22 ± 2.21	1 (11.1)	2 (22.2)	4 (44.4)	2 (22.2)		

*Note*: V, visit.

^a^ Comparison change from baseline in V4–V6 with V3 period.

### 3.5 6MWT

**FIGURE 3:**
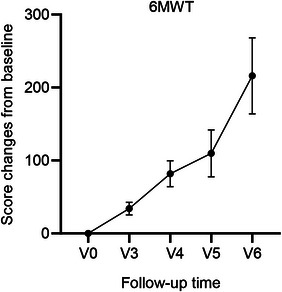
Change curve of 6‐min walk test (6MWT) scores for baseline and V3–V6 period. V, visit period.

A total of 16 children were tested for 6MWT. 6MWT at baseline was 172.38 ± 136.80 m; Visit V3 was increased to 34.06 ± 8.58 m; Visit V4 was increased to 81.85 ± 17.64 m; Visit V5 was increased to 109.75 ± 32.14 m; and Visit V6 was increased to 216.00 ± 52.08 m. The improvement was greater as the treatment time was extended (Figure 3).

### 3.6 Factors influencing HFMSE scores at V4

**FIGURE 4:**
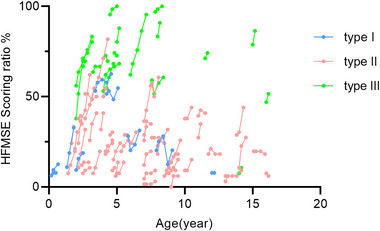
Longitudinal representations of percentages of maximum points on motor scales at V0, V4, and V5 for all spinal muscular atrophy (SMA) subtypes. HFMSE, Hammersmith Functional Motor Scale Expanded. Type, SMA Types 1–3.

Factors that may influence HFMSE scores, such as age at treatment, gender, baseline HFMSE, SMN2 copy number, and SMA clinical typing, were analyzed. A significant difference was found in HFMSE scores in children who started treatment before the age of 5 years and those who started treatment after the age of 5 years (*p* < .05), indicating that HFMSE scores improved more rapidly in children who started treatment before the age of 5 years (Figure 4). In addition, the HFMSE scores at baseline were divided into four groups based on their values: less than 10, 10–20, 20–40, and greater than 40. There was no statistical difference among the four groups. In addition, there was no statistical difference in the improvement of HFMSE scores by SMN2 copy number, clinical type of SMA, and gender (Table 5). The results suggest that age at the time of nusinersen treatment has a predictive value for improvement in HFMSE.

**TABLE 5:** Effect of different factors on the change of Hammersmith Functional Motor Scales Expanded (HFMSE) from baseline during V4 period.

Variable	Level	Coefficient	95% confidence interval	*p* value
Age of treatment initiation	<5 vs. ≥5	2.464	0.608–6.054	.018
Gender	Male vs. female	−0.516	−3.600 to 2.131	.608
Baseline HFMSE	0 ≤ V0 < 10 vs. 10 ≤ V0 < 20	0.139	−2.831 to 3.243	.891
	0 ≤ V0 < 10 vs. 20 ≤ V0 < 40	−1.016	−6.112 to 2.069	.319
	0 ≤ V0 < 10 vs. 40 ≤ V0 < 60	0.349	−3.526 to 4.937	.731
Copy number of SMN2	2 vs. 3	−0.055	−4.416 to 4.180	.956
SMA clinical typing	Type 2 vs. Type 3	−1.442	6.116–1.011	.156

### 3.7 Adverse event

A total of 805 intrathecal injections were performed, and none of the children had their treatment interrupted due to failure of a lumbar puncture. All patients underwent routine blood, urine, faecalis, biochemical and coagulation tests, as well as routine cerebrospinal fluid and biochemical tests, before intrathecal injection. No abnormalities in biochemical parameters were detected in the children. One child with SMA1 had severe pneumonia prior to nusinersen sodium treatment and was admitted to our ICU. After three treatments with nusinersen sodium, there was some improvement in limb strength, but the lung infection did not resolve, and he died of severe lung infection and respiratory failure. Eight children had had one to two episodes of lumbago or back pain, all of which lasted and resolved within 3 days of the lumbar puncture. Five children presented with vomiting, all transient and lasting 1 day in remission, and five children presented with upper respiratory tract infection, pulmonary infection, and gastrointestinal infection after puncture. One case of hypothermia appeared, lasted for 1 day, and resolved itself. Alopecia was seen in three cases, one 12‐year‐old child with severe alopecia that lasted for 1 month and resolved on its own, one child aged 1 year and 2 months with only slight alopecia, and another child aged 16 years with alopecia occurring from V3 to V4; during this period, the child basically stopped eating rice and noodles in order to lose weight and lost about 10 kg in 4 months, so the child's alopecia was not ruled out as a result of weight loss, and after resuming a normal diet, the hair loss was significantly reduced. There was one case of an 11‐month‐old child who developed a facial rash on the evening of the third treatment, and the rash lasted for 4–6 h before resolving on its own (Table 6). Most of the adverse reactions occurred during the loading period; a few adverse reactions, such as back pain, vomiting, and infection, can also occur during the maintenance period.

**TABLE 6:** Adverse events (AEs) after lumbar injections.

	*N* = 84
Total AE	26
Dizziness	1
Back pain	8
Nausea	1
Vomiting	5
Diarrhea	1
Abdomen pain	1
Low fever	1
Upper airway infection	2
Pneumonia	2
Gastroenteritis	1
Alopecia	3
Rash	1

## 4 DISCUSSION

A Phase II, open‐label, dose‐escalation study showed a mean increase of 11.66 points in CHOP INTEND score after nusinersen sodium treatment (Finkel et al., 2021). A single‐center study from Hungarian showed a 14.9 (±5.1) point increase in CHOP INTEND score in SMA1 patients, a 7.2 point increase in HFMSE score from baseline for SMA Type 2 patients, and a 4.2 point increase in RULM score from the baseline period (Szabó et al., 2020). A two‐center clinical study of the efficacy of nusinersen sodium in 61 pediatric and adult SMA patients over a 14‐month period showed that 43/59 (72.9%) of patients treated with nusinersen sodium showed improvement in their exercise scale after 14 months of treatment (Osredkar et al., 2021). A Swiss multicenter observational study at 6–41 months showed that 41 pediatric and adult SMA patients treated with nusinersen had an increase in CHOP INTEND score at last follow‐up of 2–42 points from baseline, with a median of 25 points (Tscherter et al., 2022), and mean HFMSE scores at 6, 10, and 14 months after starting treatment were significantly higher compared to baseline. In a retrospective observational clinical study of nusinersen treatment of adult SMA patients, clinically meaningful improvements (≥3 points) in HFMSE scores were noted in 35 of 124 patients (28%) at 6 months, 33 of 92 (35%) at 10 months, and 23 of 57 (40%) at 14 months (Hagenacker et al., 2020). A multicenter, double‐blind, placebo‐controlled trial found that 57% of children in the nusinersen group, compared to 26% in the control group, had an increase of at least 3 points in Hammersmith Functional Motor Scale Expanded (HFMSE) scores at the 15th month compared to baseline (Mercuri et al., 2018). A prospective study from Polish similarly noted an 8.9 point increase in CHOP INTEND score compared to baseline after 12 months of nusinersen treatment in 298 children with 5qSMA (Lefeuvre et al., 2022). The study by Darras et al. (2019) also demonstrates that nusinersen treatment improves motor function and stabilizes disease activity in patients with later‐onset spinal muscular atrophy (SMA), including Type 3. Our study also found significant improvements in HFMSE scores, CHOP INTEND scores, RULM scores, and 6MWT in children with SMA at Stages V4–V6 after Nusinersen Nason therapy.

Our study found a negative correlation between the age of initiation of treatment and the clinical efficacy of nusinersen. The younger the child, the better the relative treatment outcome; this may be due to the effects of muscle contracture, weight, and scoliosis. Two papers based on studies of the natural disease process in SMA noted that the risk of a decline in HFMSE score of more than two points at 12 months increased with age at baseline (Calucho et al., 2018). In contrast, children aged less than 5 years had stable or spontaneous improvement in scores without intervention. A prospective study in Poland showed a negative correlation between the age at initiation of treatment and the CHOP Intend score at baseline and the clinical outcome of nusinersen; that is, the younger the age at initial treatment, the better the motor function reserve at baseline and the better the treatment response to nusinersen (Kotulska et al., 2022). Belančić et al. (2023) found that in SMA Type 3 patients who commenced nusinersen treatment at the age of 18 or older, there was no improvement in Revised Hammersmith Scale (RHS) and 6MWT. This is generally consistent with the results of our study.

No correlation between SMN2 copy number and clinical outcome was found in this study. This is inconsistent with the study by Osredkar et al. (2021), who concluded that SMN2 copy number was positively correlated with improvement in HFMSE scores after treatment. The possible reason is that most children in that study had two or three copies of SMN2 and very few had four or one copy number. Calucho et al. (2018) showed that in 625 Spanish SMA patients, 2 (43%) or 3 copies of the SMN2 gene (46%) were present in most patients, and their genotype–phenotype correlation was not very clear. Three copies of SMN2 were detected in patients with all three major SMA types, with the vast majority being Type 2 or 3 cases (57% and 37%, respectively). Their study suggests that genotype and phenotype are not identical if the child has two or three copies of SMN2, and that one or more copies are more predictive of clinical phenotype. As the main therapeutic target of nusinersen is SMN2, the predictive value of SMN2 copy number for nusinersen treatment becomes limited when other modifier genes are at play. This was confirmed in a study by Tscherter et al. (2022), who concluded that SMN2 copy number did not correlate with improvements in motor function.

There are some limitations in this study. This study is a retrospective clinical efficacy observational study rather than a prospective cohort study, with a relatively small sample size, and an even smaller sample size when the study population is divided into subgroups, which will affect the assessment of the results; due to the late entry of nusinersen into the Chinese market and the limited follow‐up observation time, the long‐term clinical effects and adverse effects of nusinersen were not observed. The above conclusions need to be confirmed by prospective, multicenter, large sample size, and long‐term observational studies.

## 5 CONCLUSION

Through a retrospective clinical efficacy observation of a two‐center nusinersen treatment for SMA in western China, we found that the CHOP INTEND score, HFMSE score, and RULM score were significantly higher in children after treatment compared to baseline. There was a negative correlation between nusinersen age and clinical outcome of nusinersen, with younger age being associated with better response to treatment. No serious adverse effects affecting treatment and quality of life were observed during nusinersen treatment for SMA.

## AUTHOR CONTRIBUTIONS

**Dan Li**: Conceptualization; writing—original draft. **Jie Yang**: Methodology; data curation. **Xueying Wang**: Formal analysis. **Lin Yang**: Data curation; investigation. **Rong Luo**: Methodology; validation; formal analysis. **Shaoping Huang**: Conceptualization; methodology; writing—review and editing; project administration.

### PEER REVIEW

The peer review history for this article is available at https://publons.com/publon/10.1002/brb3.3630.

## Data Availability

The data that support the findings of this study are available from the corresponding author upon reasonable request.
